# The role of knowledge and medical involvement in the context of informed consent: a curse or a blessing?

**DOI:** 10.1007/s11019-022-10121-z

**Published:** 2022-11-01

**Authors:** Caterina Milo

**Affiliations:** Robinson College, University of Cambridge, Grange Rd, Cambridge, CB3 9AN UK

**Keywords:** Informed consent, Clinical involvement, Ethics of knowledge, Prenatal testing, Reproductive ethics

## Abstract

Informed consent (IC) is a key patients’ right. It gives patients the opportunity to access relevant information/knowledge and to support their decision-making role in partnership with clinicians. Despite this promising account of IC, the relationship between ‘knowledge’, as derived from IC, and the role of clinicians is often misunderstood. I offer two examples of this: (1) the prenatal testing and screening for disabilities; (2) the consent process in the abortion context. In the first example, IC is often over-medicalized, that is to say the disclosure of information appears to be strongly in the clinicians’ hands. In this context, knowledge has often been a *curse* on prospective parents. Framing information in a doctor-centred and often negative way has hindered upon prospective parents’ decision-making role and also portrayed wrong assumptions upon disabled people more widely. In the second context, information is more often than not dismissed and, in a de-medicalized scenario, medical contribution often underplayed. The latter leads to an understanding of the dialogue with clinicians as a mere hinderance to the timely access to an abortion. Ultimately, I claim that it is important that knowledge, as derived from IC, is neither altogether dismissed via a process of de-medicalization, nor used as a *curse* on patients via a process of over-medicalization. None of the two gives justice to IC. Only when a better balance between medical and patients’ contribution is sought, knowledge can aspire to be a *blessing* (i.e. an opportunity for them), not a *curse* on patients in the IC context.

## Introduction

There is wide consensus in the legal and ethical debate concerning the significance of informed consent (IC) as a key patients’ right. The latter entails that before any medical decision is achieved the disclosure of information is vital. This is also because IC can be used as a means to give patients access to relevant knowledge and hence to support their decision-making role in partnership with clinicians. Despite this promising account of IC, what seems to be less discussed is the relationship between ‘knowledge’, as derived from IC, and the role of clinicians. In my reflection, I will claim that knowledge should be framed as an opportunity *for* patients, not a burden/curse to be inflicted upon them by clinicians. However, the tie between knowledge and medical involvement has been often misunderstood. I offer two examples of this: (1) the prenatal testing and screening for disabilities; (2) the consent process in the abortion context. In the first example, IC is often over-medicalized, that is to say the disclosure of information appears to be strongly in the clinicians’ hands. In this context, knowledge has often been a *curse* on prospective parents. Framing information in a doctor-centred and often negative way has hindered upon prospective parents’ decision-making role and also portrayed wrong assumptions upon disabled people more widely. In the second context, information is more often than not dismissed and, in a de-medicalized scenario, medical contribution often underplayed. The latter leads to an understanding of the dialogue with clinicians as a mere hinderance to the timely access to an abortion. Rightly framed, IC has a strong potential to be an opportunity for patients to be made aware of relevant and tailored information (i.e. access to knowledge), so as to avoid the feeling of making a ‘blind choice’ (i.e. without such opportunity). Ultimately, I claim that it is important that knowledge, as derived from IC, is neither altogether dismissed via a process of de-medicalization, nor used as a *curse* on patients via a process of over-medicalization. None of the two gives justice to IC. Only when a better balance between medical and patients’ contribution is sought, knowledge can aspire to be a *blessing* (i.e. an opportunity for them), not a *curse* on patients in the IC context.

## The tie between IC and knowledge: the ‘Power’ to ‘Empower’ patients?

IC is powerful. What does that actually mean? The existence of a patient’s right to be made aware of relevant information before a medical treatment/intervention is undergone is indeed something of key importance for patients. As it has been widely discussed in the ethical and legal debate IC has the ‘power to empower’ patients, rebalancing often a knowledge gap that they might or might not have, or more simply, putting them in a position of potential greater ownership of their medical choices (i.e. autonomy). Looked more closely, IC is hence ‘powerful’ in as far as this is tied with the opportunity to gain or delve deeper into relevant ‘knowledge’ before a medical decision is achieved.

In this context, and as the UK Supreme Court judgment in *Montgomery v Lanarkshire Health Board [2015]* also highlights, dialogue between patients and clinicians becomes the gate for a ‘right to know’ (Cave and Reinach 2019; Arden 2017) to be unpacked, namely a right to receive the information necessary to make a medical choice. In this context, and as post-Montgomery professional guidelines (PG) on consent have rightly highlighted (e.g. GMC 2020) the key to unlock this opportunity for the patient is communication with clinicians. The latter needs to be open, non-directive nor judgmental, yet truthful and tailored to the circumstances of the actual patient. This understanding of IC does not mean to say that this is ‘the’ way through which patients gain information, but instead ‘an’ important way for them, an opportunity that cannot be unilaterally dismissed without impacting on their decision-making journey.

IC is hence best framed as no longer or not merely the tool through which medical professional can ‘shake off’ their legal and ethical responsibilities, nor as a tool to ‘patronize patients’, but more so as a chance for patients to know what they are signing up for. This doesn’t mean assuming ignorance on the side of patients nor full knowledge on the doctors’ side. It conversely means, as I have also argued elsewhere (Cave, Milo, 2020), that it is *in partnership with* and *not in opposition* to the clinicians that the decision-making process can unfold.

The power of IC is hence crucially in its tie with access to relevant ‘knowledge’. The latter is to be tailored in a patient-centred way, namely in a way that is understandable and meaningful for the actual patient. Of course, the opposite is also true, namely that patients have the opportunity to refuse such knowledge and embrace a right *not to* know (Montgomery, at 85). This right however does not diminish the power of IC and its tie with knowledge, yet further reinstates that the main driver and direction of the journey are indeed patients, and that they can well express a desire not to unlock further information. Less information, sometimes, can be already more or enough for some patients.

IC, as tied to knowledge, has hence a strong potential to operate *for* patients and to support them in the decision-making process within the medical encounter with clinician(s) and beyond.

## IC and knowledge in context: two case studies

Having explored the potential of IC as linked to knowledge, the following sections will be putting this framework into context, exploring the challenges that this idea can often encounter in medical practice. I will claim that knowledge and IC can risk becoming either a *curse* upon patients in an over-medicalization of information, or can risk being rapidly dismissed by clinicians in a de-medicalized framework. Both an over-medicalization of IC and, its opposite, de-medicalization of IC, are problematic. It is true that the leading UK Supreme Court judgment on IC, i.e. *Montgomery*, wouldn’t exclude as a matter of principle neither of the two scenarios since the balance between medical and patients’ contribution is still left crucially open (Cave, Milo 2020).^1^ However, the risk of both de-medicalization and over-medicalization is that they can contradict what IC, as tied to knowledge, is or at least should aspire to be: IC should be framed as an opportunity *for* patients and *not against* them, something to be achieved in partnership *with* clinicians and not in isolation *from* them (i.e. de-medicalization) or giving clinicians a predominant role (i.e. over-medicalization).

***The over-medicalization of IC: the case of prenatal screening and testing.***

***Is knowledge a ‘curse’ on patients?***

The first way in which IC, as linked to knowledge, can be misinterpreted is through an over-medicalization of the information disclosure process. With the latter term I intend that a process of disclosure of information is primarily led by an imbalanced focus on doctors’ expertise, with little, if any weight devoted to patients’ needs and desires. The reason why I claim it is important to focus on over-medicalization is because it can show a failure within the IC process. If it is true that patients have gained more and more opportunities to access an increasing number of information, this has not necessarily been coupled with a greater sense of ownership of their decision-making process, especially when a strong medical ownership of the disclosure process has been in place. Over-medicalization has hence risked silencing patients’ autonomy and with it the safeguard of their right to IC. The example that this section is providing is the context of prenatal screenings and testing for disabilities. Through this case study I will claim that when knowledge is framed in a strong doctor-centred way (i.e. over-medicalized), there is an evident risk of framing IC as a *curse* on patients rather than an opportunity *for* them.

Recent years have seen a widespread opportunity for prospective parents to access prenatal screening and testing during the pregnancy journey. It is particularly the case that access to non-invasive prenatal testing (NIPT),^2^ has clearly opened up an even wider range of chances for prospective parents to ‘know’ potentially more. But is this always framed in an ‘empowering way’ for them? In other words, is the disclosure of a wider amount of information providing a real support in the decision-making process or a *curse* on prospective parents?

Already before the advent of NIPT, as Williams et al. (2002) highlights, there seemed to be a paradox here whereby the expansion in available information was not necessarily coupled with an experience of ‘enhanced choice’ or better more knowledge (Di Mattei et al. 2021; Farrell et al. 2021; Seven et al. 2016). Particularly, participants to a 2002 study on prenatal screening, though recognizing in principle the opportunity that prenatal screening could offer them through the expansion in available information, did not see themselves as necessarily better informed, but often experienced a sense of lack of sufficient knowledge (Williams 2002). A similar pattern was also reported after NIPT (Di Mattei et al. 2021). One key challenge in navigating the ‘ocean’ of information has been the often over-medicalization of information. Knowledge in the disability context has more often than not been framed as a *curse* (Gould 2020; Robinson 2019) in the hands of clinicians. If this is partially to be attributed to the technical, and sometimes complex, nature of available information, this is also due to the prominent role that clinicians have exercised in the disclosure process. Medical staff has often pushed patients firstly towards getting screened/tested, repeatedly portraying this as ‘the right course of action’. Evidence, also coming through a 2013 UK-Parliamentary enquiry (Bruce 2013), has indeed shown that women have been often blamed by clinicians for refusing to undergo prenatal screening once a disabled child was born (Williams et al. 2002; Beck-Gernstein 2000). Once a screening/testing results have been released the pressure under which prospective parents have been put often hasn’t stopped. Clinicians have repeatedly offered terminations (Shakespeare 1998) as ‘the’ course of action. It should be clarified that this paper is not saying that an expansion of information equates per se over-medicalization, nor that this is the only factor affecting the possible lack of knowledge/lack of enhanced choice on the side of patients, but that over-medicalization is one key challenge to IC that has often been unexplored in literature. What this section wants to highlight is that when the disclosure process is framed in an unbalanced way between clinicians and patients, patients’ right to IC risk being silenced. This is because an over-medicalized approach clearly trumps over the call to listen and dialogue with patients, beyond any assumptions from the clinicians’ side. The latter is not only or not merely a question of ‘amount’ of disclosure, nor of the nature of the information per se, but also of framing rightly the role that both clinicians and patients can play in the disclosure of information process.

On a more wider scale, information concerning screening and testing for disabilities has often been subject to what can be defined as a ‘medical gaslighting’, with a strong framing effect in the clinicians’ hands. This has been often translated into the provision of negative information (Gould 2020; Guon et al. 2014) concerning the medical risks connected to raising a child with a disability, with little if any room for a wider look, beyond its pure clinical terms, at the experiential reality of a disability for both children and parents. The latter, though not in the strictly medical remit of clinical expertise, is still interconnected with the disclosure process and asks clinicians to at least avoid making assumptions on behalf of the parents and/or offer opportunities for a wider social support during the decision-making process. Such informative challenges seem also to be due to what Gould calls a ‘culpable ignorance’ from the clinical side, that is to say a lack of necessary knowledge about the experiential reality of families with disabled children. Despite this lack of sufficient knowledge and/or lack of necessary trainings, the approach has been often to take control of the information sharing process in what has been defined as a ‘tyranny of medical expertise’ and provision of one-sided information (Williams 2002).

It is the case that ‘knowledge’ has been stripped away of its nature as an opportunity *for* patients and jailed prospective parents into an inextricable amount of information they do not necessarily desire. What the above-mentioned example wants to highlight is that when IC is framed in an over-medicalized way, patients’ decision-making role risk being often shrank and the true nature of IC as a supportive mechanism forgotten. A more balanced approach would seek to ensure that clinicians are made aware of relevant medical and social advances as these can positively impact upon patients’ decision-making journey, provided that the disclosure is tailored in a way that is significant for them and hence upholds their autonomy.

***The de-medicalization of IC: the abortion context and the progressive irrelevance of the medical contribution in the disclosure process***

If knowledge is often used as a weapon in the clinicians’ hand, particularly in the context of disability and prenatal screening and testing, the opposite approach of de-medicalization is neither something to be praised. This section is offering the consent process in the abortion context as an example of the dismissive role of IC, in its tie with knowledge, that clinicians have often embraced. Crucially, when the contribution of clinicians’ is reduced to the point of risking being altogether excluded, this doesn’t lead necessarily to better outcomes. In other words, when information is exclusively in the hands of the patient alone and the contribution of clinicians is made progressively irrelevant, IC, in its tie with knowledge, is not wholly safeguarded. This is because both clinical and patients’ expertise matter and it is in dialogue between them that a balance can and should be achieved.

A clear example of the de-medicalization of IC is the consent process in the abortion context in England and Wales. It might seem prima facie out of place, especially in the domestic context, to speak about IC and abortion in such terms. Formally abortion is strongly medicalized. For a legal abortion to be carried out, the Abortion Act (AA) s.1. requires two registered medical practitioners (RMPs) to authorize it in good faith. However, medical practice has also shown that the contribution of clinicians in the disclosure process has not been always taken seriously. While it is well known that IC finds theoretical support in relevant PG on abortion and consent (RCOG 2011, 2015, 2020; NICE 2019), the disclosure of information process has been often jeopardised in practice. Both the Department of Health and Social Care, DHSC (2014) and the Care Quality Commission, CQC (2016) have reported the existence of ‘dismissive’ practices on the side of clinicians. One such example being the practice of pre-signing consent forms^3^ (DHSC 2013). In this context RMPs (particularly the second RMP) have been found signing consent forms relying solely on their colleagues’ assessments and without an actual encounter with the patient. In 2016, in particular, an investigation held at Marie Stopes International abortion facilities in England raised also concerns about patient consent and the respect of the requirement of good faith. The CQC highlighted in this respect that:

Clinicians were reportedly bulk-signing HSA1 forms, which meant that they did not necessarily have access to all relevant information or sufficient time to review it before authorising a termination. Also, there was no process in place for ensuring HSA4 forms were submitted to the Department of Health within the legal timeframe of 14 days.^4^

The above-mentioned report led the CQC issuing warning notices to Marie Stopes International and the temporary suspensions of their service.

This dismissive approach risk framing the contribution of medical professional in general as progressively irrelevant within IC. Though the practice of pre-signing consent form was not altogether illegal, given that the DHSC interpretation of the Act does not require clinicians to have an actual consent encounter with a woman^5^ (DHSC 2014, at para 6), it is in a more-wide scale approach a testament of the misguided understanding of IC. The message that seems to be sent is one of irrelevance of the medical contribution in the disclosure process in abortion procedures.

This is more so nowadays where the contribution of clinicians has been further reduced and de-medicalization enhanced. In March 2022, in the aftermath of the COVID-19 pandemic, an amendment to the AA (Health and Care Bill 71, 2022) allowed telemedical access to an early term medical abortion, making permanent the changes temporarily approved in March 2020 (DHSC 2020). I have argued elsewhere (Milo 2022) that the use of telemedicine, while not excluding altogether the possibility to embrace an IC process, has at least reduced the opportunities for a medical encounter and delivered once again a message of the irrelevance of clinical involvement in the IC process. In this context, the opportunity of a dialogue with clinicians during the decision-making process seems to be more unilaterally framed as often *irrelevant*, or worse as an obstacle to the timely access to an abortion (Milo 2022).

While a reduction of medical involvement might save ‘time’, it might risk missing the broader picture concerning IC, particularly in its tie with a knowledge that is there *for* the patient. It is true that the patient can, as said above, refuse information and exercise a right not to know, however in a post-pandemic context this cannot be preventively excluded via a unilateral reduction of opportunities for this dialogue to be unpacked (as it is in a process of de-medicalization).

IC can be a gate that opens up access to wider knowledge, namely an opportunity for patients in partnership with clinicians. However, when such opportunities for a dialogical encounter with clinicians are significatively reduced, as it is in a de-medicalized context, this gate risks being left shut and patients’ needs silenced.

## Conclusion: can knowledge ever be an opportunity *for* patients?

There is a vast literature on the role of IC and clinical involvement. The uniqueness of this paper stands in its focus on the remarkably opposite roles that clinicians have played in different medical arenas. This paper makes the case for showing how in some areas clinicians have taken a strongly active role (i.e. over-medicalization) whereas in others they have had an often dismissive approach (i.e. de-medicalization) in the information disclosure process. The aim of this paper has been to show that neither of the two has given justice to how IC is and should be framed, particularly since both approaches jeopardise patients’ involvement.

Knowledge should hence be neither a *curse* on patients, bombarding them with excessive medical information that they may not necessarily find relevant for their decision-making process, nor should be altogether excluded when framed as an *obstacle* to the timely access to healthcare services. I have argued that knowledge, in a nutshell, should neither be over-medicalized nor de-medicalized.

Knowledge can and should be an opportunity *for* and not against patients.

What this means is that firstly, clinicians should refrain from making *prima facie* assumptions on what the right course of action for patients is. Especially when providing prenatal screenings and testing, the over-medicalization of information has dangerous ripple effects upon the decision-making role of prospective parents and can send wider negative messages upon the disabled population. Secondly, clinicians should neither dismiss altogether the relevance of their contribution in the disclosure-process, in a de-medicalized approach. The progressive reduction of opportunities for a dialogical encounter between clinicians and patients, particularly in a telemedical context, risks dismissing the importance of finding a truly balanced approach. IC is built also upon the role and expertise of *both* clinicians and patients. In all, the over-medicalization of information doesn’t find a solution in a de-medicalized approach, but with seeking a better balance between the contribution of patients and clinicians. The latter is often the result of a practical judgement that should be arising *through* and *from* a dialogue between the parties. While the line cannot be drawn preventively, the importance of offering opportunities for an encounter with clinician(s) should be further highlighted, particularly in a post-pandemic context, and a better balance between face-to-face and telemedicine should be also sought at the policy level.

Knowledge can and should be an opportunity for patients, when the relationship between clinical and patients’ needs and expertise finds a better balance in the medical encounter. Only then can knowledge aspire to be a *blessing*, not a *curse*.

## Footnotes

1 I have argued elsewhere with Cave that Montgomery leaves open the risk of a ‘Bolam-legacy’ i.e. of a doctor-centred approach. See: Cave et al. (2020).

2 For a definition of NIPT and analysis of the ethical challenges connected to NIPT see: Nuffield Council on Bioethics (2017).

3 RMPs need to sign what are technically called ‘notification forms’, namely HSA1–HSA2–HSA4, so as to fulfil the requirement of the Abortion Regulations 1991. For an overview of the required forms and respective guidance see: DHSC, (2013), https://www.gov.uk/government/publications/abortion-notification-forms-for-england-and-wales (accessed 25th June 2022).

4 The Care Quality Commission (CQC) has published the reports of its inspections of Marie Stopes International from earlier this year’, 20th December 2016, https://www.cqc.org.uk/news/releases/cqc-publishes-inspection-reports-marie-stopes-international (accessed 25th June 2022).

5 Although there is no legal requirement for at least one of the certifying doctors to have seen the pregnant woman before reaching a decision about a termination, the Department’s view is that it is good practice for this to be the case’, DHSC (2014, 5, para 6).
