# Correction to: The role of knowledge and medical involvement in the context of informed consent: a curse or a blessing?

**DOI:** 10.1007/s11019-022-10130-y

**Published:** 2023-02-13

**Authors:** Caterina Milo

**Affiliations:** Robinson College, University of Cambridge, Grange Rd, Cambridge, CB3 9AN UK

**Correction to: Medicine, Health Care and Philosophy** 10.1007/s11019-022-10121-z

In the original publication, the article title has been published incorrectly and reference Milo (2022) has been missed. The same has been corrected and provided in this correction.

**Reference** Milo, C. 2022. Love as a journey in the informed consent context: Legal abortion in England and Wales as a case study. *The New Bioethics* 28 (3): 208–222.

Also “this gate risk” in the last paragraph of section “**IC and knowledge in context: two case studies**” should be “this gate risks” and “clinical patients” in the last paragraph of **Conclusion** part should be “clinical and patients”.

The original article has been corrected.

